# Global trends and knowledge evolution of tertiary lymphoid structures in cancer immunotherapy: a bibliometric and visualization analysis

**DOI:** 10.3389/fimmu.2026.1907300

**Published:** 2026-09-30

**Authors:** Yanan Li, Zhi Cao, Jingru Zhou, Yang Zhang, Weidong Han, Zhiqiang Wu

**Affiliations:** 1 Department of Bio-therapeutic, The First Medical Centre, Chinese People’s Liberation Army (PLA) General Hospital, Beijing, China; 2 Changping Laboratory, Beijing, China; 3 National Clinical Research Centre for Hematologic Diseases, The First Affiliated Hospital of Soochow University, Suzhou, China

**Keywords:** antitumor immunity, bibliometrics, cancer immunotherapy, tertiary lymphoid structures (TLSs), tumor microenvironment

## Abstract

**Background:**

Tertiary lymphoid structures (TLSs) are ectopic immune aggregates that arise in non-lymphoid tissues during chronic inflammation, including cancer. Their reported associations with antitumor immunity and immune-checkpoint blockade have prompted rapid growth of the field. We therefore mapped the literature on TLSs in cancer immunotherapy over the prespecified period from January 1, 2000 through March 31, 2026.

**Methods:**

The primary bibliometric corpus comprised 1,373 English-language articles and reviews from the Web of Science Core Collection (WoSCC). PubMed and Scopus were searched independently for descriptive comparison and were not merged with the WoSCC corpus. Analyses used bibliometrix, VOSviewer, CiteSpace, SCImago Graphica, and Microsoft Excel.

**Results:**

Publication output rose markedly after 2019 and reached 315 publications in 2025. China, the United States, and France were the leading corresponding-author countries/regions, while INSERM, Sun Yat-sen University, and Fudan University led the distinct-document institutional count. Major mapped themes included B cells, immune-checkpoint blockade, spatial pathology, prognostic modeling, the tumor microenvironment, lymphatic vessels, and high endothelial venules. The independently retrieved PubMed and Scopus datasets showed broadly similar aggregate temporal and title/abstract term profiles.

**Conclusion:**

The analysis describes the growth, collaboration structure, and thematic development of TLS research in cancer immunotherapy. These bibliometric patterns indicate research attention; they do not establish biological mechanisms, biomarker performance, therapeutic efficacy, or clinical utility.

## 1 Introduction

Cancer immunotherapy has reshaped the treatment landscape for a wide range of malignancies by restoring or enhancing antitumor immune responses (1, 2). Strategies such as immune checkpoint blockade (ICB), adoptive cell therapy, and cancer vaccines have produced durable clinical benefits in a subset of patients (3–5). However, the overall response rate remains limited, and both primary and acquired resistance continue to pose major clinical challenges (6). Therefore, a better understanding of the tumor immune microenvironment (TIME) is urgently needed, along with the identification of reliable biomarkers and immune structures that can reflect effective antitumor immunity and predict therapeutic outcomes (6, 7).

Tertiary lymphoid structures (TLSs) are ectopic lymphoid aggregates that develop in non-lymphoid tissues in response to persistent inflammatory stimuli, such as chronic infection, autoimmune disease, and cancer (8, 9). Unlike secondary lymphoid organs (SLOs), TLSs form locally within affected tissues and may contain T-cell zones, B-cell follicles, follicular dendritic cells (FDCs), germinal centers, plasma cells, dendritic cells, and high endothelial venules (HEVs) (8, 10). In tumors, TLSs are increasingly recognized as specialized immune microenvironments. They support local antigen presentation, lymphocyte recruitment, B-cell maturation, and T-cell activation, thereby contributing to coordinated antitumor immune responses (11, 12).

In recent years, TLSs have received increasing attention in cancer immunotherapy research (8). Accumulating evidence suggests that the presence, maturation status, spatial organization, and cellular composition of TLSs are associated with favorable prognosis and improved responses to immune checkpoint blockade (ICB) (13–16). In particular, TLS-associated B-cell responses, tumor-infiltrating lymphocytes (TILs), dendritic cell activation, high endothelial venules (HEVs), and their interactions within the tumor microenvironment (TME) have become important topics in translational cancer immunology (17–19). These findings suggest that TLSs are not simply passive histological structures, but active immune niches that help shape antitumor immunity and therapeutic outcomes.

Given the rapid growth of this field, a systematic overview of the global research landscape, major contributors, intellectual foundations, and emerging hotspots of TLS research in cancer immunotherapy is necessary (8, 20). Bibliometric analysis provides a quantitative and visualization-based approach to examine publication trends, collaborative networks, influential journals, highly cited references, and evolving research themes (21). By combining performance analysis with science mapping, this approach can help trace the development of a research field over time and identify potential directions for future investigation (22). Similar bibliometric approaches have also been applied to immunotherapy research in specific tumor types (23).

A related bibliometric study by Bao et al. mapped the broader TLS literature from 2014 to 2023 using WoSCC and similar visualization tools (56). The present work should therefore be viewed as an incremental, cancer-immunotherapy-specific update rather than the first bibliometric study of TLSs. Its narrower disease-treatment focus, prespecified window through March 31, 2026, explicit separation of the primary and comparison datasets, and additional descriptive cross-database comparison define the scope of the contribution.

In this study, WoSCC served as the sole primary bibliometric corpus. PubMed and Scopus were searched independently for descriptive cross-database comparison; the three datasets were analyzed separately and were not merged. Using the 1,373-record WoSCC corpus, we examined publication and citation trends, journals, corresponding-author countries/regions, institutions, authors, co-cited references, keyword networks, thematic clusters, timelines, and citation bursts. The comparison datasets were used only to assess broad aggregate temporal and title/abstract term patterns.

## 2 Materials and methods

### 2.1 Data source and search strategy

The database search was conducted on April 1, 2026. WoSCC was used as the sole primary data source because it provides standardized bibliographic metadata and cited-reference information required for co-citation, citation-burst, and science-mapping analyses. The prespecified study window was January 1, 2000 through March 31, 2026. The initial WoSCC export generated on April 8, 2026 contained 1,381 English-language articles and reviews. After publication-date screening against the prespecified cutoff, the cutoff-aligned export dated April 10, 2026 contained 1,373 records. PubMed and Scopus were searched independently for descriptive comparison and were not pooled with, or used to calculate bibliometric indicators for, the primary WoSCC corpus. To reduce false retrieval caused by other meanings of “TLS,” the abbreviation alone was not used as an independent search term; full expressions for tertiary or ectopic lymphoid structures were used.

The search formula was as follows: TS = ((“tertiary lymphoid structure*” OR “tertiary lymphoid organ*” OR “ectopic lymphoid structure*” OR “ectopic lymphoid organ*” OR “tertiary lymphoid aggregate*”) AND (immunotherap* OR “cancer immunotherap*” OR “tumor immunotherap*” OR “immune checkpoint*” OR “immune checkpoint block*” OR “checkpoint blockade” OR “immune checkpoint inhibitor*” OR “PD-1” OR PD1 OR “PD-L1” OR PDL1 OR “CTLA-4” OR CTLA4 OR “adoptive cell therap*” OR “adoptive cell transfer*” OR “CAR-T” OR “CAR T” OR vaccin* OR “oncolytic virus*”) AND (cancer* OR tumor* OR tumour* OR neoplasm* OR malignan* OR carcinoma*)).

Only English-language articles and reviews were included. The full records and cited references were exported from WoSCC in tab-delimited text format for subsequent bibliometric analysis.

### 2.2 Inclusion and exclusion criteria

The inclusion criteria were as follows: (1) publications related to TLSs and cancer immunotherapy; (2) document types limited to articles and reviews; (3) publications written in English; and (4) records indexed in the WoSCC.

The exclusion criteria were as follows: (1) non-English publications; (2) document types other than articles or reviews, including meeting abstracts, editorial materials, letters, corrections, proceedings papers, and other non-research document types; (3) duplicate WoSCC records identified by accession number (UT); and (4) records with a publication or electronic-publication date after March 31, 2026.

Eligibility was assessed using the prespecified database, document-type, language, date-window, and topic-search criteria. Duplicate checking within the initial WoSCC export identified no duplicate UT identifiers. Eight records dated after March 31, 2026 were excluded, leaving the final primary WoSCC corpus of 1,373 records. No independent dual-reviewer title/abstract or full-text screening was undertaken, and the manuscript does not imply such a procedure. PubMed and Scopus were analyzed separately, so cross-database overlap was not treated as duplication.

### 2.3 Bibliometric analysis and visualization

Bibliometric analyses and visualizations were performed using Microsoft Excel (Microsoft Corporation, Redmond, WA, USA), R software (version 4.4.2; R Foundation for Statistical Computing, Vienna, Austria) with the bibliometrix package (version 5.3.0), VOSviewer (version 1.6.20; Leiden University, Leiden, the Netherlands), CiteSpace (version 6.4.R1; Drexel University, Philadelphia, PA, USA), and SCImago Graphica (Beta version 1.0.53; SCImago Lab).

Before analysis, country/region names, author names, journal titles, and keywords were reviewed for traceable spelling, abbreviation, and formatting variants. Institutional display labels were standardized typographically, while distinct WoS Organization-Enhanced (C3) entities were retained unless exact equivalence was documented. Journal Impact Factors (JIFs) and JCR quartiles correspond to the 2025 journal metrics released in the 2026 Journal Citation Reports (Clarivate). The same JCR release was applied consistently in **Figure 1** and **Tables 1** and **2**, and JIFs were reported to one decimal place.

**Figure 1:**
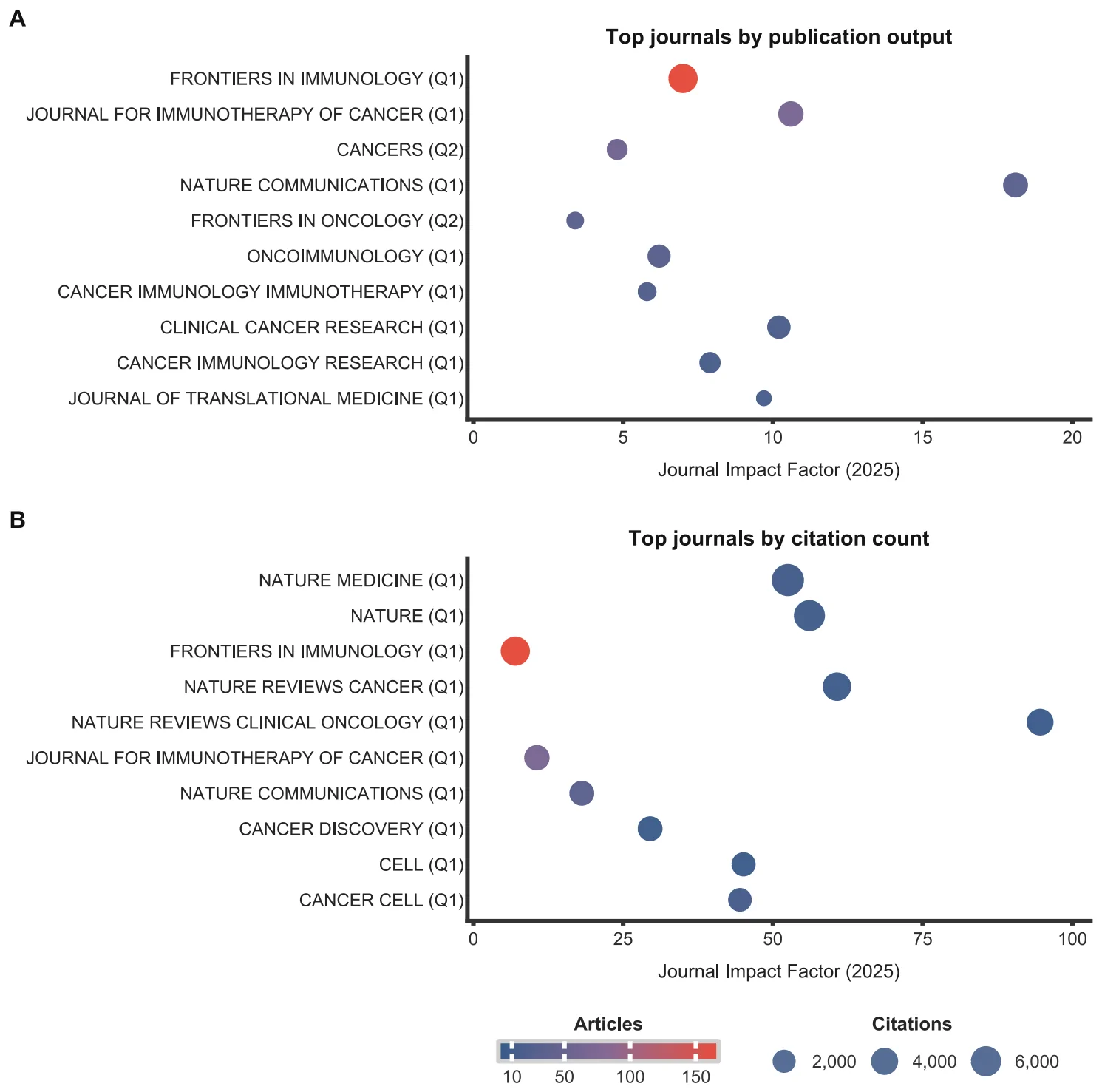
Leading journals by publication output and total citations in TLS-related cancer immunotherapy research. **(A)** The 10 journals with the highest publication output in the final 1,373-record WoSCC corpus. **(B)** The corpus-wide top 10 journals ranked by total citations. In both panels, horizontal position indicates the 2025 Journal Impact Factor (JIF), bubble size indicates the total citations accrued by the included records published in each journal, and marker color indicates publication output. Total citations were calculated as the sum of WoSCC Times Cited values for the included records assigned to each journal. JIFs and JCR quartiles correspond to the 2025 journal metrics released in the 2026 Journal Citation Reports (Clarivate); quartiles are shown in parentheses after journal names. JIF was used only as the horizontal-axis variable and did not determine the rankings in either panel.

**Table 1:** Top 20 most productive journals in TLS-related cancer immunotherapy research.

Journal	Publications (n)	Total citations (n)	JIF (2025; JCR 2026)	JCR quartile	Mean citations per publication
Frontiers in Immunology	167	5,284	7.0	Q1	32
Journal for ImmunoTherapy of Cancer	68	3,068	10.6	Q1	45
Cancers	57	1,243	4.8	Q2	22
Nature Communications	35	2,826	18.1	Q1	81
Frontiers in Oncology	34	422	3.4	Q2	12
OncoImmunology	33	2,011	6.2	Q1	61
Cancer Immunology, Immunotherapy	25	651	5.8	Q1	26
Clinical Cancer Research	18	2,132	10.2	Q1	118
Cancer Immunology Research	17	1,358	7.9	Q1	80
Journal of Translational Medicine	16	155	9.7	Q1	10
Cancer Cell	14	2,260	44.5	Q1	161
Scientific Reports	14	201	4.9	Q1	14
Translational Lung Cancer Research	14	209	3.4	Q2	15
Advanced Science	13	84	14.1	Q1	6
British Journal of Cancer	13	898	7.8	Q1	69
Cancer Medicine	13	292	3.5	Q2	22
International Journal of Molecular Sciences	13	225	5.6	Q1	17
Nature Medicine	12	7,395	52.5	Q1	616
Cancer Research	11	871	16.6	Q1	79
Cancer Science	10	141	4.9	Q2	14

JIF, Journal Impact Factor. JIF values and JCR quartiles correspond to the 2025 journal metrics released in the 2026 Journal Citation Reports (Clarivate). Publications (n) denotes included records published by the listed journal; total citations (n) denotes summed WoSCC Times Cited values at final export; mean citations per publication = total citations/publications, rounded to the nearest integer.

**Table 2:** Top 10 most cited journals in TLS-related cancer immunotherapy research.

Journal	Total citations (n)	Publications (n)	JIF (2025; JCR 2026)	JCR quartile
Nature Medicine	7,395	12	52.5	Q1
Nature	6,573	10	56.1	Q1
Frontiers in Immunology	5,284	167	7.0	Q1
Nature Reviews Cancer	4,805	9	60.7	Q1
Nature Reviews Clinical Oncology	3,816	5	94.6	Q1
Journal for ImmunoTherapy of Cancer	3,068	68	10.6	Q1
Nature Communications	2,826	35	18.1	Q1
Cancer Discovery	2,798	6	29.5	Q1
Cell	2,454	7	45.1	Q1
Cancer Cell	2,260	14	44.5	Q1

JIF, Journal Impact Factor. JIF values and JCR quartiles correspond to the 2025 journal metrics released in the 2026 Journal Citation Reports (Clarivate). Rankings are based on total citations within the final 1,373-record WoSCC corpus; JIF was not used to determine rank.

Country output in **Table 3** was generated with the bibliometrix (version 5.3.0) Corresponding Author’s Countries routine. Each record was assigned to one corresponding-author country/region, using the WoS reprint/corresponding-address field when available and C1 otherwise. SCP and MCP classify publications whose complete affiliation set involved one country/region or more than one country/region, respectively; MCP ratio equals MCP divided by publications. Institutional output in **Table 4** used distinct-document full counting from the WoS Organization-Enhanced (C3) field. Repeated occurrences of the same C3 entity within a record were counted once, while different entities were retained. These non-mutually-exclusive institution counts are not directly comparable with the corresponding-author-country counts.

**Table 3:** Top 10 countries/regions by corresponding-author publication output in TLS-related cancer immunotherapy research.

Country/region	Publications (n)	SCP (n)	MCP (n)	Corpus share	MCP ratio
China	600	560	40	0.437	0.067
United States	240	179	61	0.175	0.254
France	95	56	39	0.069	0.411
Japan	68	64	4	0.050	0.059
Germany	39	25	14	0.028	0.359
Italy	38	29	9	0.028	0.237
United Kingdom	32	16	16	0.023	0.500
Netherlands	31	13	18	0.023	0.581
Canada	24	12	12	0.017	0.500
South Korea	24	20	4	0.017	0.167

Publications are included WoSCC articles and reviews assigned using bibliometrix Corresponding Author’s Countries. SCP, single-country publications; MCP, multiple-country publications; corpus share = publications/1,373; MCP ratio = MCP/publications. These values are distinct from all-affiliation full-counting weights in **Figure 2**.

**Table 4:** Top 20 institutions by unique publication count in TLS-related cancer immunotherapy research.

Rank	Institution	Unique publications (n)
1	INSERM	103
2	Sun Yat-sen University	73
3	Fudan University	66
4	Université Paris Cité	65
5	Sorbonne University	65
6	UNICANCER	59
7	University of Texas System	57
8	Shanghai Jiao Tong University	47
9	Harvard University	42
10	MD Anderson Cancer Center	42
11	Harvard University Medical Affiliates	41
12	Nanjing Medical University	40
13	Chinese Academy of Medical Sciences–Peking Union Medical College	40
14	Assistance Publique–Hôpitaux de Paris (APHP)	37
15	Peking Union Medical College	36
16	University of California System	35
17	Gustave Roussy	34
18	Pennsylvania Commonwealth System of Higher Education	32
19	University of London	31
20	University of Pittsburgh	30

Institutional output was calculated from the WoS Organization-Enhanced (C3) field using distinct-document full counting. The same entity was counted at most once per record, while a multi-institutional record could contribute once to each represented institution. Counts are non-mutually exclusive and should not be summed or directly compared with **Table 3**. Records without a C3 entry (n = 16) did not contribute.

Microsoft Excel was used to summarize annual publication and citation trends, and bibliometrix supported descriptive journal and corresponding-author-country analyses. VOSviewer (version 1.6.20) used full counting for the country/region and institution/author co-authorship networks, cited-reference co-citation network, and keyword co-occurrence network. In **Figure 2**, node size represents document count. In **Figure 3**, the displayed item weight was Links, so node size represents the number of direct co-authorship links. In **Figure 4A** and **Figure 5A**, node size represents citation frequency and keyword occurrence frequency, respectively. Across VOSviewer maps, link width represents pairwise link strength, node color denotes cluster membership when cluster colors are displayed, and link color does not encode an additional quantitative variable. SCImago Graphica (Beta version 1.0.53) was used to render the country/region collaboration displays in **Figure 2** from the retained weighted VOSviewer GML network.

**Figure 2:**
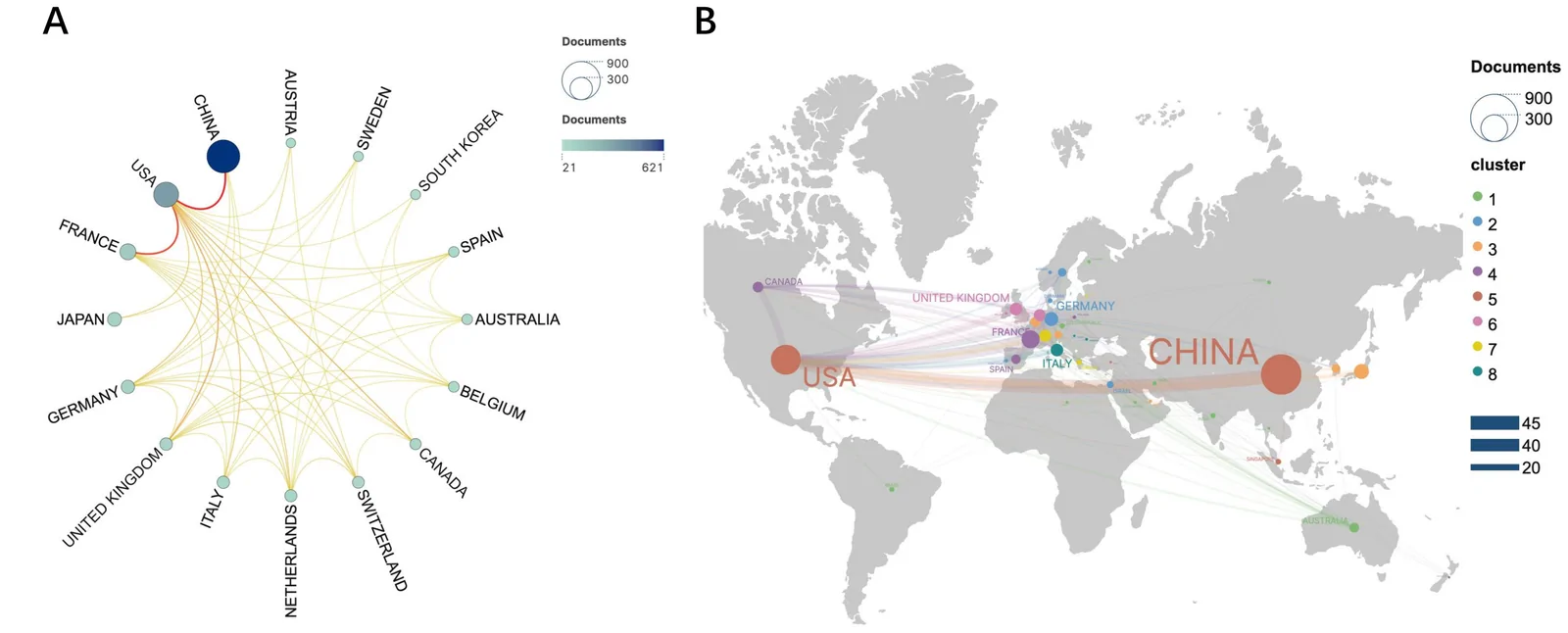
Country/region co-authorship patterns in TLS-related cancer immunotherapy research. Country/region co-authorship data from the final 1,373-record WoSCC corpus were constructed in VOSviewer (version 1.6.20) using full counting, exported as a weighted GML network, and rendered in SCImago Graphica (Beta version 1.0.53). **(A)** Circular collaboration network restricted to countries/regions with a minimum threshold of 21 documents, comprising 16 nodes and 114 links. Node size and fill intensity represent document count. Link width represents pairwise co-authorship weight. Link color is used as a display gradient and does not encode a variable independent of collaboration weight. No cluster encoding is displayed in panel **(A)**. **(B)** Geographic collaboration map of the complete retained GML network, with no additional display threshold, comprising 43 nodes and 302 links. Node size represents document count, and link width represents pairwise co-authorship weight. Node colors indicate the eight VOSviewer cluster assignments stored in the source GML. Link colors follow the displayed cluster palette and do not encode an independent quantitative variable. No re-clustering was performed in SCImago Graphica. **Figure 2** uses all listed affiliations; its country/region weights are therefore not numerically equivalent to the corresponding-author country/region counts reported in **Table 3**.

**Figure 3:**
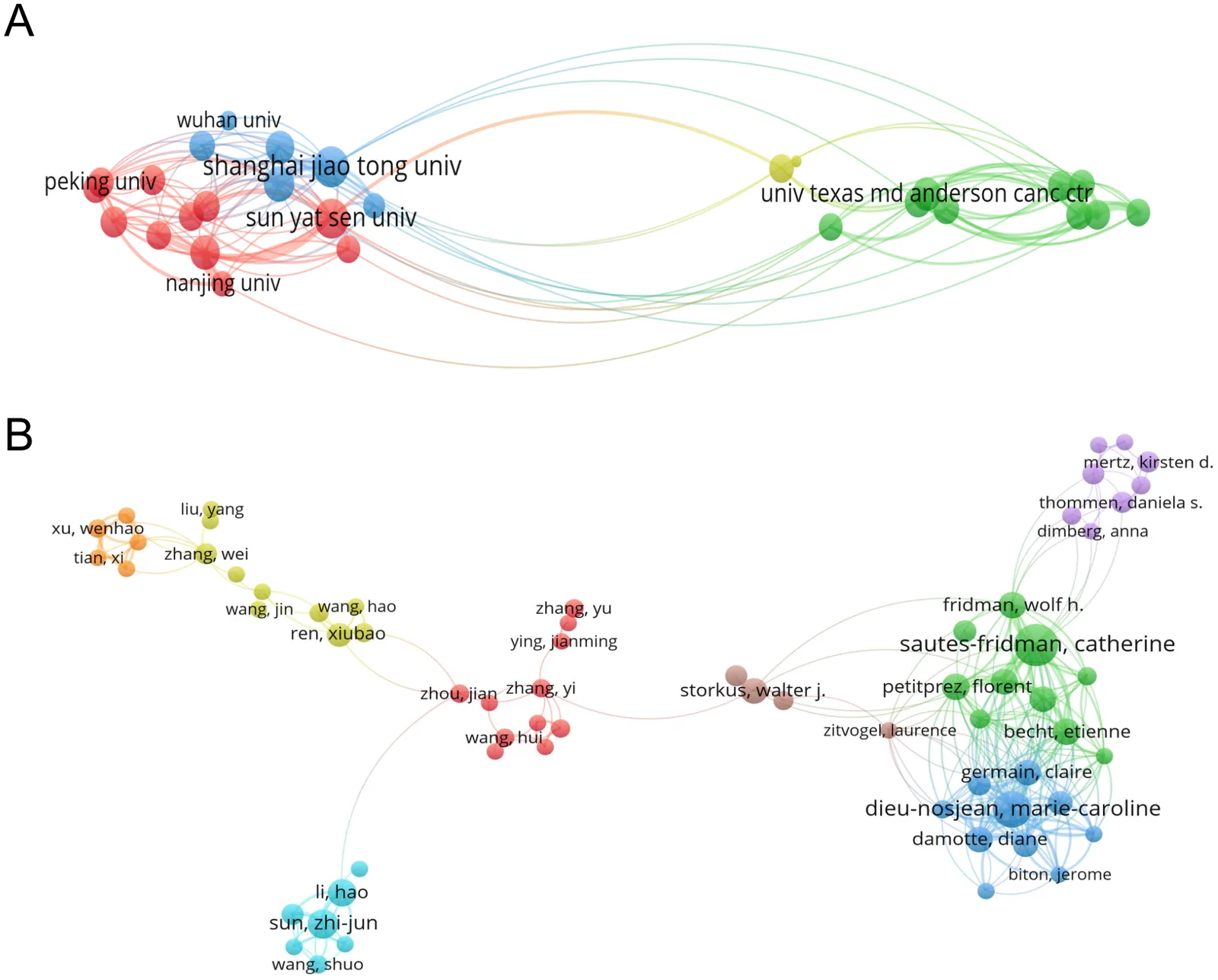
Institutional and author co-authorship networks in TLS-related cancer immunotherapy research. Both panels were generated from the final 1,373-record WoSCC corpus in VOSviewer (version 1.6.20) using full counting. **(A)** Institution-level co-authorship network. VOSviewer identified 2,028 organizations. With minimum thresholds of 17 documents and 0 citations per organization, 28 organizations met the criteria and all 28 were included in the visualization. **(B)** Author-level co-authorship network. Documents containing more than 25 authors were excluded from this network analysis. VOSviewer identified 9,485 authors. With minimum thresholds of 5 documents and 0 citations per author, 120 authors met the criteria; the largest connected component, comprising 65 authors, was displayed. In both final network visualizations, the item weight was set to Links; node size therefore represents the number of direct co-authorship links. Links indicate pairwise co-authorship relationships, and link width reflects pairwise link strength. Node colors identify clusters generated by VOSviewer’s built-in clustering procedure. Link colors follow the colors of the connected clusters and do not encode an independent quantitative variable. Curved links were used for display.

**Figure 4:**
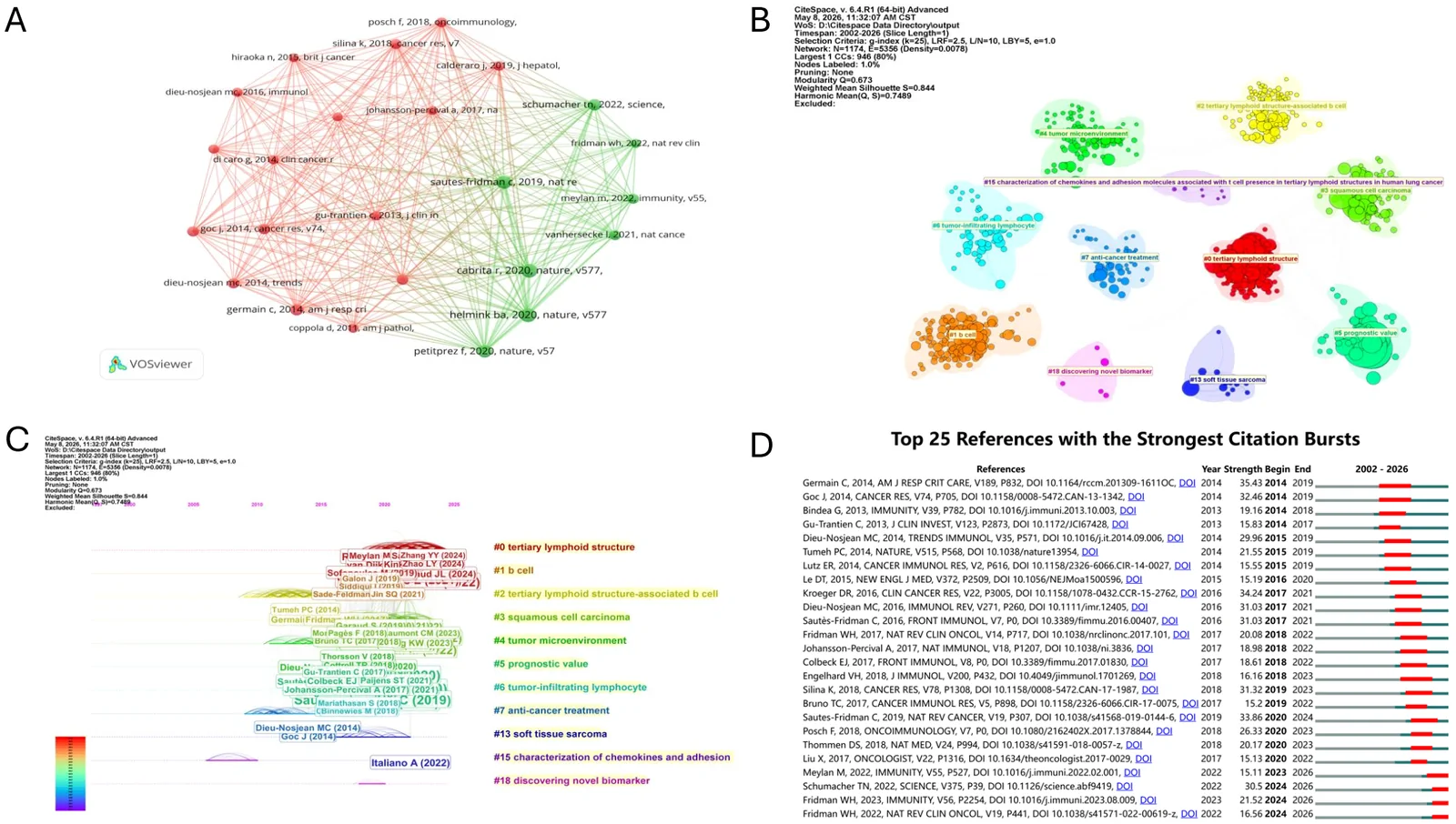
Reference co-citation structure, thematic clusters, temporal evolution, and citation bursts in TLS-related cancer immunotherapy research. All panels were based on the final 1,373-record WoSCC corpus. **(A)** The reference co-citation network was generated in VOSviewer (version 1.6.20) using full counting. A minimum citation threshold of 150 was applied; 24 cited references met the threshold and all 24 were included in the final network. Each node represents a cited reference; node size indicates citation frequency. Links indicate pairwise co-citation relationships, and link thickness indicates pairwise co-citation strength. Node colors distinguish clusters generated by VOSviewer’s built-in clustering procedure; link colors follow the cluster color scheme and do not encode an independent quantitative variable. **(B)** The co-citation cluster map and **(C)** timeline view were generated in CiteSpace (version 6.4.R1) over the observed publication span from 2002 through 2026 using 1-year time slices; the 2026 slice includes records only through March 31, 2026. Candidate nodes were selected within each annual slice using the g-index (k = 25), with LRF = 2.5, L/N = 10, LBY = 5, and e = 1.0; 1.0% of nodes were labeled and no pruning was applied. The VOSviewer full-versus-fractional counting distinction is not applicable to these CiteSpace panels; their inclusion rule was the per-slice g-index. The network contained 1,174 cited-reference nodes and 5,356 co-citation links (density = 0.0078); the largest connected component contained 946 nodes (80%). Node size indicates citation frequency, links indicate co-citation relationships, and link thickness indicates co-citation strength. In panel B, colors distinguish the identified co-citation clusters; in panel C, cited references are arranged by cluster along the time axis, and node and link colors follow the annual time-slice color scale. CiteSpace spectral clustering yielded modularity Q = 0.673 and a weighted mean silhouette S = 0.844; cluster labels were generated using latent semantic indexing (LSI). **(D)** The 25 references with the strongest citation bursts were identified using CiteSpace burst detection. The light-blue baseline indicates the complete observation period, whereas red segments indicate the detected burst intervals. Strength denotes burst intensity and Begin and End denote the starting and ending years of each burst.

**Figure 5:**
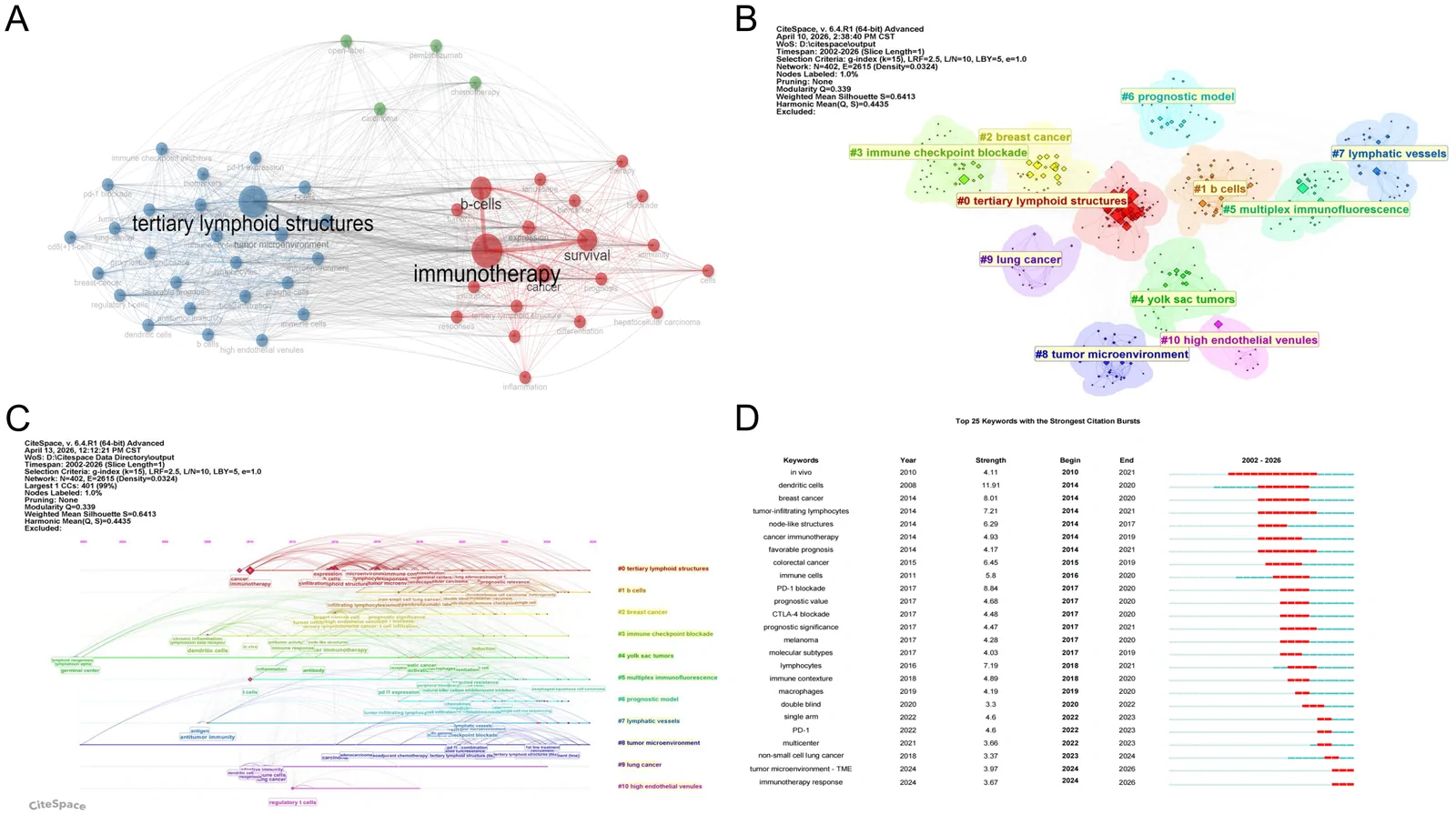
Keyword co-occurrence structure, thematic clusters, temporal evolution, and burst analysis in TLS-related cancer immunotherapy research. All panels were based on the final 1,373-record WoSCC corpus. **(A)** The keyword co-occurrence network was generated in VOSviewer (version 1.6.20) using full counting. The unit of analysis was all keywords, comprising Author Keywords and Keywords Plus terms analyzed as source strings. A minimum occurrence threshold of 42 was applied; 50 keywords met the threshold and all 50 were included in the final network. Each node represents a keyword; node size indicates occurrence frequency. Links indicate pairwise keyword co-occurrence, and link thickness indicates pairwise co-occurrence strength. Node colors distinguish clusters generated by VOSviewer’s built-in clustering procedure; link colors follow the cluster color scheme and do not encode an independent quantitative variable. **Table 6** is a separate source-string frequency summary and is not a direct listing of the VOSviewer node-occurrence weights shown in panel **(A)**. **(B)** The keyword co-occurrence cluster map and **(C)** timeline view were generated in CiteSpace (version 6.4.R1) over the observed publication span from 2002 through 2026 using 1-year time slices; the 2026 slice includes records only through March 31, 2026. Title, abstract, Author Keywords (DE), and Keywords Plus (ID) fields were enabled. Candidate nodes were selected within each annual slice using the g-index (k = 15), with LRF = 2.5, L/N = 10, LBY = 5, and e = 1.0; 1.0% of nodes were labeled and no pruning was applied. The VOSviewer full-versus-fractional counting distinction is not applicable to these CiteSpace panels; their inclusion rule was the per-slice g-index. The resulting network contained 402 keyword nodes and 2,615 co-occurrence links (density = 0.0324); the largest connected component contained 401 nodes (99%). Node size indicates keyword occurrence frequency, and link thickness indicates co-occurrence strength. In panel B, colors distinguish the 11 keyword clusters (#0-#10). CiteSpace spectral clustering yielded modularity Q = 0.339 and a weighted mean silhouette S = 0.6413; cluster labels were generated using latent semantic indexing (LSI). In panel C, keywords are arranged by cluster along the time axis, and node and link colors follow the annual time-slice color scale. **(D)** The 25 keywords with the strongest bursts were identified using CiteSpace burst detection. The light-blue baseline indicates the complete observation period, whereas red segments indicate the detected burst intervals. Strength denotes burst intensity, and Begin and End denote the starting and ending years of each burst.

The final VOSviewer selections were frozen as follows. **Figure 2A** used a minimum of 21 documents per country/region and displayed 16 nodes and 114 links; **Figure 2B** displayed the complete retained 43-node, 302-link GML network without an additional display threshold. **Figure 3A** used minimum thresholds of 17 documents and 0 citations per organization; 28 of 2,028 organizations met the criteria and all 28 were displayed. **Figure 3B** excluded documents with more than 25 authors, used minimum thresholds of 5 documents and 0 citations per author, and displayed the largest connected component of 65 authors among 120 threshold-eligible authors identified from 9,485 authors. **Figure 4A** used a minimum of 150 citations and displayed all 24 eligible cited references. **Figure 5A** used all keywords (Author Keywords and Keywords Plus), a minimum occurrence threshold of 42, and displayed all 50 eligible keywords. These values are the documented final settings used to generate the revised panels. They are reported as controlled final visualization settings rather than as retrospective recovery of every historical submission-era run; no sensitivity analysis across alternative VOSviewer counting, threshold, or clustering settings was performed.

CiteSpace (version 6.4.R1) used annual slices from 2000 through 2026; the 2026 slice contains records only through March 31. The reference co-citation analysis used g-index selection with k = 25, and the keyword analysis used k = 15. Both used LRF = 2.5, L/N = 10, LBY = 5, e = 1.0, 1% node labeling, and no pruning. CiteSpace spectral clustering partitioned the networks, and latent semantic indexing (LSI) generated cluster labels. The reference network contained 1,174 nodes and 5,356 links (density = 0.0078; modularity Q = 0.673; weighted mean silhouette S = 0.8440); the keyword network contained 402 nodes and 2,615 links (density = 0.0324; Q = 0.339; weighted mean S = 0.6413). Automated labels and moderate silhouette values were treated as exploratory rather than as biological validation. The display settings for the journal dual-map overlay are reported in the **Figure 6** legend.

**Figure 6:**
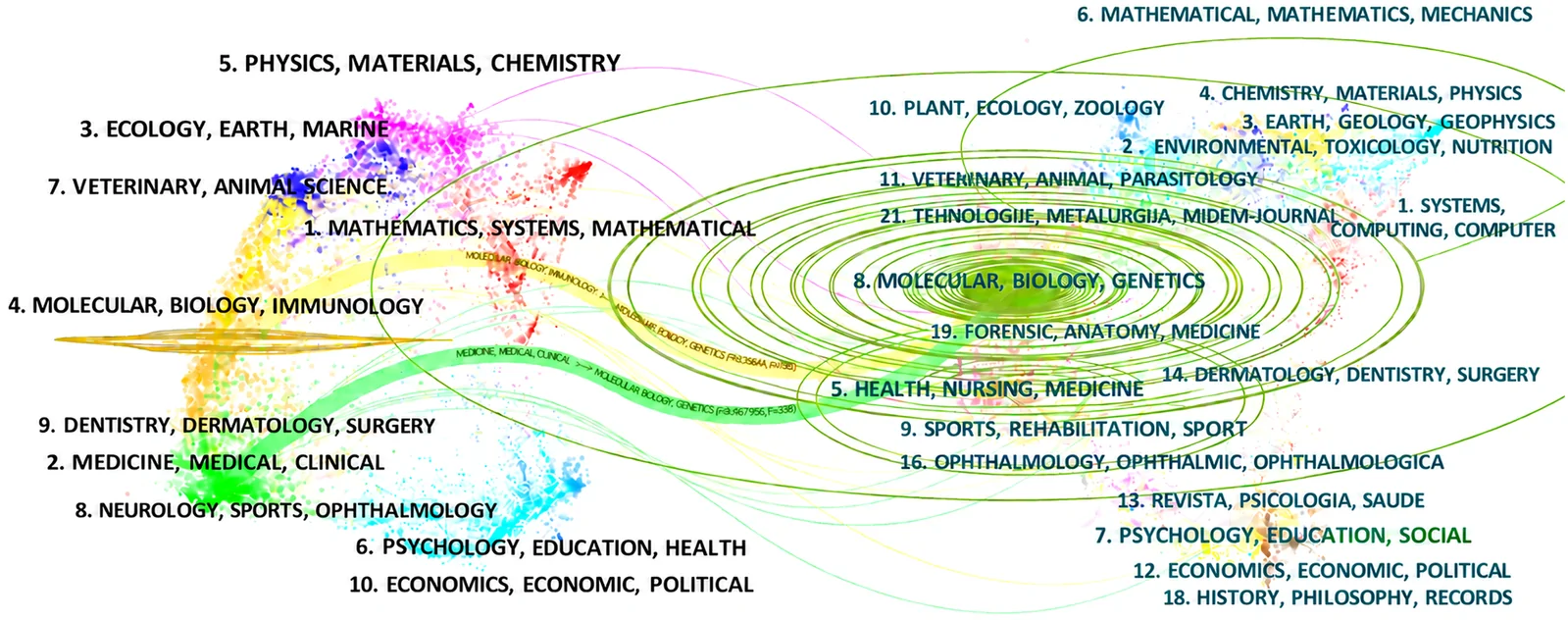
Dual-map overlay of citing and cited journals in TLS-related cancer immunotherapy research. The dual-map overlay was generated in CiteSpace (version 6.4.R1) from the final 1,373-record WoSCC corpus covering January 1, 2000 through March 31, 2026. The fixed base map on the left represents citing-journal disciplinary domains, whereas the fixed base map on the right represents cited-journal disciplinary domains. Curves indicate citation trajectories from citing to cited disciplinary domains. Trajectory color identifies the originating citing-journal domain, and trajectory width reflects the relative magnitude of the displayed citation flow with z-score scaling enabled. The final display settings were Cluster Labels = 24 with log transformation enabled; Journal Labels = 0; Arcs alpha = 3; Citing Articles >= 10 with a scaling parameter of 1.0; Cited Times >= 100 with a scaling parameter of 0.25; Snap to Centroids (<Radius) = 0; and z Scores enabled. The disciplinary communities displayed on the base maps used the Blondel option shown in the CiteSpace dual-map interface. Because this visualization projects the bibliographic corpus onto predefined journal-domain base maps rather than constructing an item-selected node network, conventional full versus fractional counting, a node-level inclusion threshold, a study-specific included-node count, and node-size encoding are not applicable. The value 1,373 refers to the number of input bibliographic records, not to the number of network nodes. The numbered disciplinary-domain labels were enlarged for readability; the underlying base map and citation trajectories were not changed.

Counts are reported as integers, descriptive percentages and JIFs to one decimal place, **Table 3** corpus shares and MCP ratios to three decimal places, **Table 5** silhouette values to two decimal places, and years as integers. “Publications” denotes included records assigned under the stated table-specific rule; “total citations” denotes summed WoSCC Times Cited values at final export; “SCP” and “MCP” denote single-country and multiple-country publications, respectively; and MCP ratio equals MCP divided by publications. **Table 6** is a separate descriptive summary of Author Keywords (DE) and Keywords Plus (ID) source strings. Each exact source string was counted at most once per record and counts were then summed for the listed label. For the first row, the singular and plural forms “tertiary lymphoid structure” and “tertiary lymphoid structures” were combined; other spelling, spacing, and hyphenation variants remained separate unless explicitly stated. These values are source-string totals rather than normalized concept counts, unique-document counts, or VOSviewer node-occurrence values.

**Table 5:** Major keyword clusters in TLS-related cancer immunotherapy research.

Cluster ID	Size (n)	Silhouette value	Mean year	Automated LSI label	Interpretation
#0	66	0.55	2018	Tertiary lymphoid structures	Core knowledge base of TLS research in cancer immunotherapy
#1	51	0.61	2022	B cells	Recent B-cell and TLS-maturation theme
#2	43	0.59	2017	Breast cancer	Automated disease-related label; interpret with representative terms
#3	43	0.82	2013	Immune checkpoint blockade	Earlier cluster; not evidence of a recent cluster-level shift
#4	38	0.61	2015	Yolk sac tumors	Automated disease-related label; interpret conservatively
#5	38	0.57	2021	Multiplex immunofluorescence	Recent spatial-pathology and TLS-characterization theme
#6	35	0.56	2022	Prognostic model	Recent prognostic and translational theme
#7	30	0.69	2018	Lymphatic vessels	Structural microenvironment related to TLS formation
#8	30	0.67	2021	Tumor microenvironment	Recent local immune-ecology theme
#9	15	0.90	2011	Lung cancer	Earlier automated disease-related label; interpret conservatively
#10	12	0.93	2013	High endothelial venules	Earlier vascular-organization theme

Cluster labels were generated automatically by CiteSpace using latent semantic indexing (LSI). Labels summarize terms represented within each cluster and should be interpreted together with cluster size, silhouette value, mean year, and representative terms. Automated labels do not independently establish the biological, clinical, temporal, or field-level importance of a topic.

**Table 6:** Top 20 keyword labels by summed source-string occurrence count in TLS-related cancer immunotherapy research.

Rank	Listed keyword label	Summed source-string occurrences (n)
1	tertiary lymphoid structure(s)	860
2	immunotherapy	753
3	b-cells	433
4	survival	416
5	cancer	327
6	expression	224
7	tumor microenvironment	219
8	t-cells	205
9	dendritic cells	191
10	prognosis	180
11	tumor-infiltrating lymphocytes	128
12	responses	125
13	regulatory t-cells	113
14	cells	102
15	immunity	96
16	open-label	91
17	chemotherapy	90
18	lymphocytes	88
19	plasma-cells	88
20	tumor	86

**Table 6** is a descriptive source-string frequency summary derived from the Author Keywords (DE) and Keywords Plus (ID) fields and should not be interpreted as a direct listing of the VOSviewer node-occurrence weights in **Figure 5A**. Frequency (n) is the sum of record-level occurrences of the source string or explicitly combined source strings represented by each listed label. An exact source string was counted at most once per record. For “tertiary lymphoid structure(s),” the singular (n = 140) and plural (n = 720) source strings were summed (n = 860); because 19 records contained both forms, this value is not a unique-document count. Other spelling, spacing, and hyphenation variants remained separate unless explicitly stated. These values are source-string totals, not normalized concept counts.

### 2.4 Cross-database comparison

To examine whether broad aggregate temporal and title/abstract term patterns were represented in other databases, PubMed and Scopus were searched independently with database-specific syntax adapted from the WoSCC concept framework. PubMed used MeSH and Title/Abstract terms; Scopus used TITLE-ABS-KEY. PubMed retrieved 967 records; 17 records carrying the publication-type label Preprint were excluded, leaving 950. Scopus yielded 1,151 records. No combined corpus, record-level matching, overlap analysis, or cross-database deduplication was performed. Direct annual and thematic comparisons were restricted to complete calendar years 2000–2025 because 2026 coverage and cutoff procedures were not equivalent; the corresponding subsets were WoSCC n = 1,287, PubMed n = 880, and Scopus n = 1,032. For the thematic comparison, normalized title-plus-abstract text was evaluated with one prespecified database-independent, human-readable term dictionary covering 12 topics. Each record contributed at most one hit per topic, and topics were not mutually exclusive. The analysis was descriptive and was not intended as formal external validation. Detailed searches and counting rules are provided in **Supplementary Methods S1, S2** and **Supplementary Tables S1, S2**.

## 3 Results

### 3.1 Literature retrieval and screening

The literature retrieval and screening process is shown in **Figure 7**. The initial WoSCC export contained 1,381 English-language articles and reviews. No duplicate WoS accession numbers (UTs) were identified within that export. Eight records with publication or electronic-publication dates after March 31, 2026 were excluded, yielding the cutoff-aligned final WoSCC primary corpus of 1,373 records and 1,373 distinct UT identifiers. All primary bibliometric and knowledge-mapping analyses used this corpus. The prespecified window was January 1, 2000 through March 31, 2026; no eligible publications occurred in 2000 or 2001, and 2002 was the first represented year. PubMed yielded 967 records, of which 17 preprint-indexed records were excluded, leaving 950; Scopus yielded 1,151 records. These comparison datasets were analyzed separately from WoSCC.

**Figure 7:**
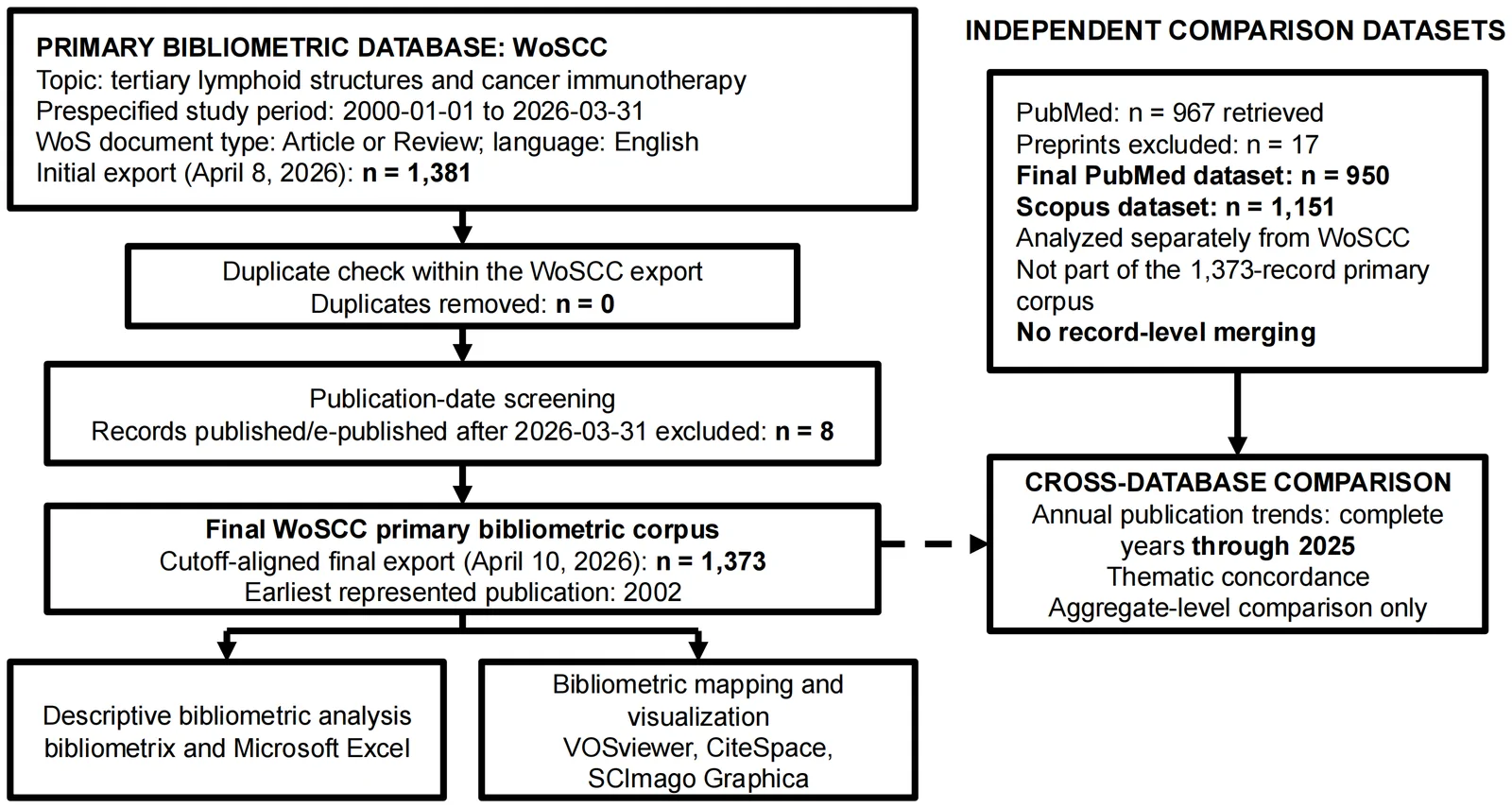
Study design, construction of the primary WoSCC bibliometric corpus, and independent cross-database comparison. The prespecified eligibility window was January 1, 2000 through March 31, 2026. The initial WoSCC export dated April 8, 2026 contained 1,381 English-language articles or reviews. Duplicate checking within this export identified no duplicate Web of Science accession numbers (UTs). Eight records with publication or electronic-publication dates after March 31, 2026 were excluded, yielding the cutoff-aligned final WoSCC primary corpus of 1,373 records (final export dated April 10, 2026); the earliest represented publication was from 2002. This corpus was used for all primary descriptive bibliometric and knowledge-mapping analyses. Descriptive analyses were performed using bibliometrix and Microsoft Excel, and mapping and visualization used VOSviewer, CiteSpace, and SCImago Graphica. PubMed yielded 967 records, of which 17 records indexed with the publication type Preprint were excluded, leaving 950; Scopus yielded 1,151 records. PubMed and Scopus were analyzed separately as descriptive comparison datasets, were not merged with WoSCC at the record level, and did not contribute to the 1,373-record primary corpus. Cross-database annual and thematic comparisons were restricted to complete calendar years through 2025 and were interpreted at the aggregate level.

### 3.2 Annual publication and citation trends

**Figure 8** shows annual publication and citation trends across the prespecified period from January 1, 2000 through March 31, 2026. No eligible publications were identified in 2000 or 2001; 2002 was therefore the first non-zero publication year. Research output remained low before 2014, increased gradually from 2014 onward, and rose more rapidly after 2019.

**Figure 8:**
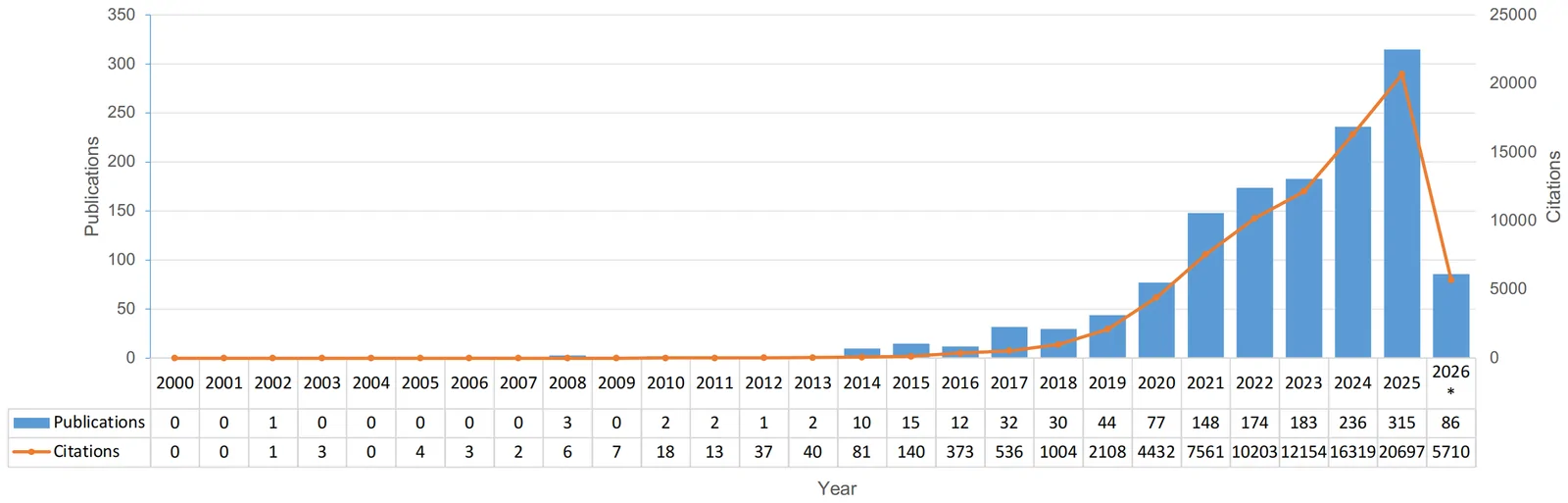
Annual publication and citation trends in TLS-related cancer immunotherapy research. The bars show annual publication counts from the final 1,373-record WoSCC primary corpus (left y-axis), and the line shows the annual citation series retained from the original Web of Science citation analysis (right y-axis). The prespecified study window was January 1, 2000 through March 31, 2026. No eligible publications occurred in 2000-2001, and 2002 was the first represented year. The highest complete-year values were observed in 2025, with 315 publications and 20,697 citations. The 2026 values (86 publications and 5,710 citations) cover January 1 through March 31 only and should not be interpreted as a full-year decline. Citation points are joined by straight segments; no smoothing or fitted trend line was applied.

Publication output reached its highest complete-year level in 2025, with 315 publications, while annual citations reached 20,697. The 2026 values (86 publications and 5,710 citations) cover January 1 through March 31 only and should not be interpreted as a full-year decline.

### 3.3 Journal distribution and citation impact

The distribution of leading journals is shown in **Figure 1** and **Table 1**. Frontiers in Immunology was the most productive journal, with 167 publications and 5,284 citations, followed by Journal for ImmunoTherapy of Cancer (68 publications; 3,068 citations) and Cancers (57; 1,243). JIFs and quartiles in **Figure 1** and **Tables 1** and **2** correspond to the 2025 journal metrics released in the 2026 Journal Citation Reports and were applied consistently across the two tables.

Several journals with fewer included publications accumulated high citation totals. Nature Medicine contributed 12 publications and 7,395 citations (mean 616 citations per publication). Cancer Cell contributed 14 publications and 2,260 citations. These figures describe citation impact within the included WoSCC corpus and should not be interpreted as clinical evidence.

The top 10 most cited journals are listed in **Table 2**. Nature Medicine ranked first with 7,395 citations, followed by Nature (6,573), Frontiers in Immunology (5,284), Nature Reviews Cancer (4,805), and Nature Reviews Clinical Oncology (3,816).

### 3.4 Dual-map overlay of journals

**Figure 6** shows the dual-map overlay of journals. The left side of the map represents citing journals, whereas the right side represents cited journals. The citation trajectories showed that publications in this field were mainly distributed in journals related to molecular biology, immunology, medicine, and clinical medicine. The cited references were also mainly concentrated in journals covering molecular biology, genetics, health, medicine, and clinical disciplines.

These citation paths suggest that TLS-related cancer immunotherapy research has a multidisciplinary knowledge structure. The field is closely connected with tumor immunology and molecular biology, as well as clinical oncology and translational medicine.

### 3.5 Country and regional contributions

**Figure 2** shows all-affiliation collaboration patterns, whereas **Table 3** summarizes corresponding-author-country output. Under the **Table 3** counting rule, China ranked first with 600 publications (43.7%), followed by the United States (240), France (95), Japan (68), and Germany (39).

The United States had 61 multiple-country publications (MCPs). France had 39 MCPs and an MCP ratio of 0.411. The Netherlands (0.581), the United Kingdom (0.500), and Canada (0.500) had the highest MCP ratios among the listed corresponding-author countries/regions. These values are not directly comparable with the all-affiliation network weights in **Figure 2**.

The country/region collaboration network further showed that China and the United States held central positions in this field. Strong collaborative links were observed between China and the United States, as well as among major European countries, including France, Germany, Italy, the United Kingdom, and the Netherlands. These findings suggest that TLS-related cancer immunotherapy research has formed a broad international collaboration network, mainly spanning Asia, North America, and Europe.

### 3.6 Institutional and author collaboration networks

**Table 4** lists institutions using distinct-document full counting after within-record deduplication. INSERM ranked first with 103 unique publications, followed by Sun Yat-sen University (73), Fudan University (66), Université Paris Cité (65), Sorbonne University (65), and UNICANCER (59). Because a multi-institutional record can contribute once to each represented institution, the counts are non-mutually exclusive and should not be summed or compared directly with **Table 3**. In **Figure 3A**, 28 organizations met the minimum threshold of 17 documents and were displayed. In **Figure 3B**, 120 authors met the minimum threshold of 5 documents after excluding documents with more than 25 authors; the largest connected component of 65 authors was displayed.

The institutional collaboration network contained several visually distinct clusters. Chinese institutions, including Fudan University, Sun Yat-sen University, Central South University, Zhejiang University, Nanjing Medical University, Shanghai Jiao Tong University, and the Chinese Academy of Medical Sciences-Peking Union Medical College, were prominent within the displayed network. Institutions from the United States and Europe formed additional collaboration groups, indicating a multi-regional co-authorship structure.

The author collaboration network displayed several closely connected research groups. Prominent nodes included Catherine Sautès-Fridman, Wolf H. Fridman, Florent Petitprez, Jérôme Goc, Claire Germain, Maxime Meylan, Isabelle Cremer, and Diane Damotte. These patterns identify co-authorship structure and should not be interpreted as a direct measure of scientific quality or causal influence.

### 3.7 Co-cited references and intellectual structure

**Figure 4** presents the reference co-citation analysis. In **Figure 4A**, 24 cited references meeting the minimum citation threshold of 150 formed a dense VOSviewer co-citation network. The displayed references primarily concerned the identification and characterization of TLSs in tumors, prognostic associations, TLS-associated B-cell responses, immune-checkpoint blockade, and antitumor immunity.

**Figure 4B** shows the cluster map of co-cited references generated by CiteSpace. The main clusters included “tertiary lymphoid structure,” “B cell,” “tertiary lymphoid structure-associated B cell,” “squamous cell carcinoma,” “tumor microenvironment,” “prognostic value,” “tumor-infiltrating lymphocyte,” “anti-cancer treatment,” “soft tissue sarcoma,” and “discovering novel biomarker.” These clusters indicate that the major research topics in this field are closely related to TLS biology, B-cell immunity, tumor immune infiltration, prognosis, and treatment response.

**Figure 4C** shows the timeline view of the co-cited-reference clusters. Earlier clusters emphasized the characterization and prognostic associations of TLSs, whereas later portions of the timeline contained increasing attention to TLS maturation, B-cell responses, tumor-microenvironment organization, and immunotherapy-related questions. This is a temporal pattern in the cited literature, not evidence that the mapped topics themselves are biologically or clinically validated.

**Figure 4D** displays the 25 references with the strongest citation bursts. Earlier bursts were associated with tumor TLS identification, tumor-infiltrating lymphocytes, chemokine and adhesion-molecule expression, and prognosis. More recent bursts involved B-cell function, TLS-associated antitumor immunity, and immune-checkpoint blockade. Citation bursts indicate concentrated scholarly attention during the detected intervals and do not measure study quality or treatment efficacy.

Taken together, the co-citation, timeline, and burst results describe an evolution in the literature from early descriptive and prognostic work toward greater attention to immune mechanisms, spatial organization, and therapeutic-response questions.

### 3.8 Keyword co-occurrence and research hotspots

**Figure 5A** presents the VOSviewer keyword co-occurrence network, whereas **Table 6** provides a separate descriptive ranking of source-string totals from the Author Keywords (DE) and Keywords Plus (ID) fields. **Table 5** corresponds to the CiteSpace clusters shown in **Figures 5B–5C**, whereas **Figure 5D** presents the separate CiteSpace burst analysis. these outputs use different selection and counting procedures and are not expected to correspond row by row. In **Table 6**, the most frequent listed labels were “tertiary lymphoid structure(s)” (860), “immunotherapy” (753), “b-cells” (433), “survival” (416), and “cancer” (327). The first value is the sum of the singular and plural TLS source-string counts and is not a unique-document count. Generic or context-dependent strings were interpreted cautiously. In particular, “open-label” was retained for transparency but was not interpreted as a distinct hotspot.

The VOSviewer keyword network in **Figure 5A** included all 50 keywords meeting the minimum occurrence threshold of 42. Strong co-occurrence links connected tertiary lymphoid structures, immunotherapy, b-cells, survival, and the tumor microenvironment, indicating that these themes frequently appeared together in the indexed literature. The VOSviewer node weights should not be read directly from **Table 6** because the latter uses the separate source-string summation described above.

**Figure 5B** shows the CiteSpace keyword clusters, all of which are reported in **Table 5**: “tertiary lymphoid structures” (#0; size 66; mean year 2018), “B cells” (#1; 51; 2022), “breast cancer” (#2; 43; 2017), “immune checkpoint blockade” (#3; 43; 2013), “yolk sac tumors” (#4; 38; 2015), “multiplex immunofluorescence” (#5; 38; 2021), “prognostic model” (#6; 35; 2022), “lymphatic vessels” (#7; 30; 2018), “tumor microenvironment” (#8; 30; 2021), “lung cancer” (#9; 15; 2011), and “high endothelial venules” (#10; 12; 2013). These are automated LSI labels and were interpreted together with cluster size, silhouette, mean year, and representative terms.

**Figure 5C** presents the cluster timeline. The recent mean years of “B cells” (#1; 2022), “multiplex immunofluorescence” (#5; 2021), “prognostic model” (#6; 2022), and “tumor microenvironment” (#8; 2021) indicate increasing attention to these topics. The earlier “immune checkpoint blockade” (#3; 2013), “yolk sac tumors” (#4; 2015), and “lung cancer” (#9; 2011) clusters were not used as evidence of a recent cluster-level shift.

**Figure 5D** shows the 25 keywords with the strongest bursts. Earlier burst terms were mainly related to lymphocyte infiltration, tumor types, and prognosis, whereas several later bursts involved immune-checkpoint blockade, B-cell-related terms, spatial characterization, and immunotherapy response. These patterns indicate changes in research attention and should not be interpreted as evidence that the corresponding biomarkers or therapeutic strategies are validated.

### 3.9 Cross-database comparison

Independent searches retrieved 967 PubMed and 1,151 Scopus records. After excluding 17 PubMed records indexed with the publication type Preprint, the final PubMed comparison dataset contained 950 records. For **Figure 9**, both the annual and thematic comparisons were restricted to complete calendar years 2000-2025, comprising 1,287 WoSCC, 880 PubMed, and 1,032 Scopus records. Aggregate publication growth and the 12 prespecified title/abstract term profiles were broadly similar across the separately analyzed datasets. This descriptive concordance does not imply record-level equivalence, database interchangeability, or formal external validation.

**Figure 9:**
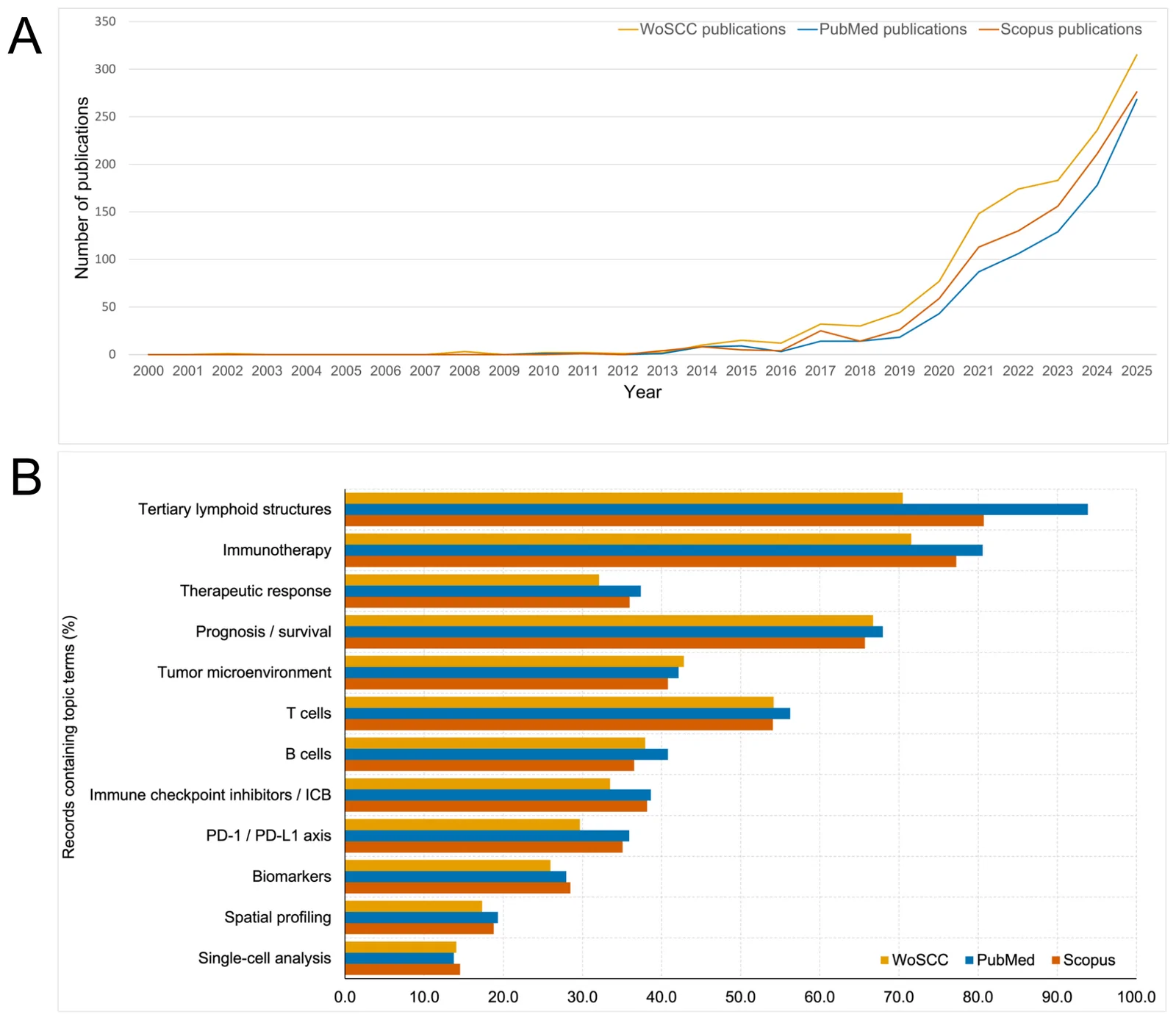
Cross-database descriptive comparison of TLS-related cancer immunotherapy research. WoSCC was the sole primary bibliometric corpus; PubMed and Scopus were independently retrieved comparison datasets. **(A)** Annual publication counts for the common complete-calendar-year window from 2000 through 2025. The plotted subsets contained 1,287 WoSCC records, 880 PubMed records, and 1,032 Scopus records. The 2026 observations were omitted because database-specific coverage and cutoff procedures were incomplete and non-equivalent. **(B)** Database-specific percentages of records containing each of 12 prespecified thematic term sets in normalized title-plus-abstract text, calculated separately within the same complete-year subsets (WoSCC n = 1,287; PubMed n = 880; Scopus n = 1,032). Each record contributed at most one count to a given topic, and topics were not mutually exclusive. The three datasets were analyzed separately; no pooling, record-level matching, overlap analysis, or cross-database deduplication was performed. The figure therefore describes aggregate temporal and thematic concordance and does not constitute formal external validation or record-level equivalence.

## 4 Discussion

### 4.1 Principal findings

This study provides a bibliometric and visual overview of TLS-related cancer immunotherapy research within the prespecified period from January 1, 2000 through March 31, 2026. Publication and citation output rose rapidly after 2019. Under the stated counting rules, China, the United States, and France led corresponding-author-country output, while INSERM, Sun Yat-sen University, Fudan University, Université Paris Cité, and Sorbonne University were the leading institutions by unique-document count. These results describe a rapidly growing and interdisciplinary literature.

Co-citation and keyword analyses identified themes involving TLS biology, B-cell immunity, immune-checkpoint blockade, the tumor microenvironment, spatial pathology, prognostic models, lymphatic vessels, and high endothelial venules. These are patterns in the mapped literature rather than mechanistic or clinical effects established by this study. Published primary studies provide the biological context for reported associations between TLSs, survival, and immunotherapy response (25, 26).

Compared with Bao et al.’s broader 2014–2023 TLS analysis (56), our study uses a narrower cancer-immunotherapy question and extends the descriptive window through March 31, 2026. The overlap in leading countries and themes is acknowledged; the contribution is an updated, domain-specific map with transparent database-role separation rather than a claim of wholly distinct or first-in-field findings.

### 4.2 From histological structures to organized immune niches

The annual trend and mapped topics indicate that the literature has expanded from histological descriptions and prognostic associations toward questions about TLS function, maturation, and spatial organization. The cited biological literature discusses TLSs as organized immune niches that may support antigen presentation, lymphocyte recruitment, B-cell maturation, and T-cell activation (20, 27); those functions were not directly tested here.

The more recent “B cells” (#1; 2022), “multiplex immunofluorescence” (#5; 2021), “prognostic model” (#6; 2022), and “tumor microenvironment” (#8; 2021) clusters indicate increasing attention to cellular context, spatial characterization, and clinically oriented analysis. The “immune checkpoint blockade” cluster (#3; mean year 2013) was not used as evidence of a recent cluster-level shift.

The cited literature indicates that TLS density, maturation, spatial distribution, cellular composition, and stromal context may influence reported biological and clinical associations (17, 29). In the present study, prominence of these topics indicates research emphasis, not proof that any TLS feature is prognostic or treatment-predictive.

### 4.3 B cells as a central theme in TLS-related cancer immunotherapy

B cells were prominent in both the source-string occurrence table and the cluster map. This bibliometric prominence identifies a major research theme but does not establish a central mechanistic role.

To contextualize this signal, cited studies report potential roles for TLS-associated B cells in antigen presentation, germinal-center-like reactions, antibody production, and interaction with T cells (10, 11, 26, 30). These mechanisms were evaluated in the cited primary studies, not in the present bibliometric analysis. This contextual evidence includes the reported association of B cells and tertiary lymphoid structures with immunotherapy response (24).

Taken together, the cited biological studies—not the bibliometric analysis itself—support context-dependent roles for B cells in TLS-related immunity. They may help explain reported associations between TLS-positive tumors, prognosis, and immune-checkpoint blockade response (31), but they do not establish a universal mechanism.

### 4.4 Spatial pathology and multiplex analysis: from counting TLSs to defining functional TLSs

The “multiplex immunofluorescence” cluster and terms related to immune-cell composition and prognosis indicate increasing use of spatially resolved methods. Cited studies illustrate the application of multiplex imaging, single-cell and spatial transcriptomics, imaging mass cytometry, digital pathology, and artificial-intelligence-assisted image analysis (32–34).

These literature-based developments may have biological and clinical relevance, but their implications were not tested by our analysis. Cited studies suggest that TLS associations may vary with maturation, spatial organization, and cellular interactions (17, 35, 36).

The mapped literature increasingly emphasizes measures beyond simple TLS counts, including maturation, location, immune-cell composition, HEV abundance, and stromal features. This prominence identifies research priorities; it does not establish the validity or predictive performance of a particular scoring system.

### 4.5 High endothelial venules, lymphatic vessels, and stromal organization

The “lymphatic vessels” and “high endothelial venules” clusters indicate research attention to tissue-level immune organization. Cited studies report associations between HEVs, lymphocyte entry, a T-cell-inflamed microenvironment, and immunotherapy response in selected settings (37–40). These biological and clinical observations derive from the cited studies, not from the present analysis.

The co-occurrence of vascular, stromal, and immune-cell topics supports their joint consideration in future primary studies. Our analysis does not test how HEVs, lymphatic vessels, stromal cells, or chemokines shape TLS function.

### 4.6 TLSs as biomarkers of prognosis and immunotherapy response

Terms related to survival, prognosis, response, and immunotherapy were frequent, and “prognostic model” formed a cluster. These patterns indicate sustained research attention to prognosis and treatment-response questions; they do not establish biomarker performance. The cited literature reports associations across several tumor types (16, 18, 28, 31, 41–45).

Whether TLS assessment adds information beyond PD-L1 expression, tumor mutational burden, microsatellite instability, and CD8^+^ T-cell infiltration remains to be established in direct comparative studies. The literature provides a rationale for prospective evaluation, not evidence of current clinical utility.

Reported TLS associations may differ by tumor type, anatomical location, maturation, treatment setting, and immune context. Standardized definitions, direct comparator analyses, multicenter validation, and prospective clinical studies are required before routine use.

### 4.7 From observation to intervention: therapeutic induction of functional TLSs

Citation-burst and timeline analyses indicate that intervention has become an emerging publication theme. This pattern does not establish the efficacy of any TLS-induction strategy.

Cited preclinical studies illustrate experimental TLS-induction approaches involving IL-33-activated ILC2s, biomaterials, and local immunomodulation (9, 46–48). These reports provide biological context but do not establish clinical efficacy.

The cited literature also implicates lymphoid chemokines in immune-cell recruitment. CXCL13-producing CD4^+^ T cells were reported during early TLS formation, and CXCL13-engineered CAR-T cells showed enhanced antitumor activity and B-cell recruitment in one preclinical study; TLS formation was not demonstrated in that CAR-T study (49, 50). These findings motivate further testing but do not establish that CXCL13-engineered CAR-T cells induce TLSs.

Therapeutic TLS induction should therefore be treated as a research hypothesis. Future studies should test whether an intervention produces mature and spatially appropriate TLSs with functional evidence, rather than merely increasing TLS-like aggregates.

### 4.8 Global research output and international collaboration

Corresponding-author-country analysis placed China first, followed by the United States and France. China had an MCP ratio of 0.067, whereas France (0.411), the Netherlands (0.581), the United Kingdom (0.500), Canada (0.500), and the United States (0.254) had higher ratios. These values describe the stated counting metric and should not be compared directly with non-mutually exclusive institution counts.

The MCP pattern indicates different levels of international coauthorship under the selected metric. It does not by itself explain the causes of those differences or the quality of the resulting research. Multicenter collaboration remains relevant for harmonizing TLS assessment and validating proposed clinical applications. The broader bibliometric literature has also examined the citation impact of international collaboration (51).

### 4.9 Journal distribution and interdisciplinary knowledge flow

The journal analysis showed that *Frontiers in Immunology*, *Journal for ImmunoTherapy of Cancer*, and *Cancers* were the most productive journals, whereas Nature *Medicine*, Nature, *Nature Reviews Cancer*, and *Nature Reviews Clinical Oncology* had the strongest citation impact. The dual-map overlay further showed that TLS-related cancer immunotherapy research connects molecular biology, immunology, medicine, clinical oncology, and translational medicine (**Figure 6**). These findings suggest that TLS research lies at the intersection of basic immunology, tumor microenvironment biology, pathology, biomarker development, and clinical immunotherapy (25).

The journal and dual-map patterns show that TLS research is distributed across immunology, oncology, pathology, and multidisciplinary journals. Citation prominence reflects influence within the literature and should not be treated as evidence of clinical validity.

### 4.10 Interpretation of disease-specific clusters

The automated disease-related labels included “breast cancer” (#2), “lung cancer” (#9; mean year 2011), and “yolk sac tumors” (#4; mean year 2015). They should not be treated as equivalent or as direct evidence of emerging disease-specific fronts.

The “yolk sac tumors” label may summarize a small, heterogeneous keyword grouping rather than a major direction. It is reported for transparency and interpreted conservatively as an algorithmic label (52).

### 4.11 Clinical and translational implications

The mapped patterns identify candidate clinical and translational questions but do not validate clinical applications. Spatial pathology and prognostic modeling remain areas for primary research, including direct comparison of TLS measures with PD-L1, tumor mutational burden, microsatellite instability, T-cell infiltration, and immune-gene signatures. Digital assessment of TLSs and therapeutic response has also been evaluated in a multicenter gastric cancer study (53).

Cited observational studies have examined TLS features in relation to immunotherapy response (16, 25, 54). These findings support prospective testing of patient-stratification value; they do not establish clinical utility or predictive accuracy.

Therapeutic TLS induction is an experimental research direction. Because reported TLS functions vary with maturation, composition, tumor type, site, and treatment context, biomarker development and induction strategies require independent biological testing and prospective clinical validation.

### 4.12 Limitations

Several limitations should be acknowledged. First, WoSCC was the sole primary bibliometric database; PubMed and Scopus were separate descriptive comparison datasets and do not eliminate coverage bias. A ten-database evidence synthesis by Zeng et al. retrieved 11,438 records, of which 6,877 (60.1%) were duplicates, illustrating both overlap and database-dependent coverage (55). In our study, the zero-duplicate statement applies only to duplicate UT identifiers within the initial WoSCC export; cross-database overlap was not assessed. Second, eligibility relied on prespecified database and metadata criteria without independent dual-reviewer title/abstract or full-text screening. Third, only English-language articles and reviews were included. Fourth, citation analyses favor older and more visible publications. Fifth, the revised panels use explicitly documented final settings and thresholds, but these controlled final settings should not be interpreted as a complete reconstruction of every historical submission-era run. Complete contemporaneous transformation logs for all normalization decisions were unavailable, and no sensitivity analysis across alternative counting, threshold, or clustering settings was performed. Automated cluster labels and source-string frequencies remain exploratory. Finally, the biological examples provide context rather than a systematic evidence synthesis, and bibliometric prominence cannot establish mechanism, biomarker validity, therapeutic efficacy, or clinical causality.

### 4.13 Conclusions and future perspectives

In conclusion, research linking TLSs with cancer immunotherapy has expanded rapidly, particularly since 2019. The mapped literature increasingly addresses B-cell immunity, TLS maturation, spatial pathology, prognostic modeling, high endothelial venules, lymphatic networks, and tumor-microenvironment organization. Collaboration, co-citation, keyword, and burst analyses describe the knowledge structure of the field; they do not establish biological mechanisms or clinical utility.

The results can help prioritize questions for future studies, including standardized TLS definitions, multicenter validation, spatial multi-omics in clinically annotated cohorts, and mechanistic testing of strategies intended to induce mature TLSs. Whether TLS features are reproducible biomarkers or whether therapeutic TLS induction improves outcomes requires independent biological and prospective clinical evidence.

## Data Availability

The primary bibliometric data were obtained from WoSCC; PubMed and Scopus provided independently retrieved descriptive comparison datasets. Database-specific search strategies, derived comparison tables, and panel-specific network parameters are included in the manuscript and Supplementary Material. The underlying database exports and processing records are available from the corresponding authors upon reasonable request, subject to database licensing terms.
