# Supplementary material for: Global trends and knowledge evolution of tertiary lymphoid structures in cancer immunotherapy: a bibliometric and visualization analysis
Source: Front Immunol. 2026 Sep 30;17:1907300. doi: 10.3389/fimmu.2026.1907300 (PMC13661904; doi:10.3389/fimmu.2026.1907300)
Supplement: Supplementary file 1 [file Table1.docx]

Supplementary Material

Supplementary Table S1. Search strategies and datasets used for cross-database comparison.

| **Database** | **Analytical role** | **Search field** | **Time span/handling** | **Language** | **Document type** | **Records** |
| --- | --- | --- | --- | --- | --- | --- |
| WoSCC | Primary bibliometric corpus | TS | Prespecified 2000-01-01 to 2026-03-31 | English | Article; Review | 1,373 total; 1,287 in 2000–2025 |
| PubMed | Independent comparison dataset | MeSH + Title/Abstract | Search window: 2000-01-01 to 2026-03-31; direct annual comparison restricted to 2000–2025 | English | Journal Article; Review | 967 retrieved; 17 preprints excluded; 950 final; 880 in 2000–2025 |
| Scopus | Independent comparison dataset | TITLE-ABS-KEY | Search-year filter: 2000–2026; direct annual comparison restricted to 2000–2025 | English | Article; Review | 1,151 total; 1,032 in 2000–2025 |

WoSCC was the sole primary bibliometric corpus. PubMed and Scopus were independent descriptive comparison datasets and were not pooled or deduplicated across databases. Direct comparison was restricted to complete calendar years 2000–2025 because an equivalent March 31, 2026 cutoff was not applied across all databases.

Supplementary Table S2. Database-specific frequencies for 12 prespecified title/abstract term categories, 2000–2025.

| **Topic** | **WoSCC, n (%) N = 1,287** | **PubMed, n (%) N = 880** | **Scopus, n (%) N = 1,032** |
| --- | --- | --- | --- |
| Tertiary lymphoid structures | 907 (70.5%) | 826 (93.9%) | 833 (80.7%) |
| Immunotherapy | 921 (71.6%) | 709 (80.6%) | 797 (77.2%) |
| Therapeutic response | 413 (32.1%) | 329 (37.4%) | 371 (35.9%) |
| Prognosis / survival | 859 (66.7%) | 598 (68.0%) | 678 (65.7%) |
| Tumor microenvironment | 551 (42.8%) | 371 (42.2%) | 421 (40.8%) |
| T cells | 697 (54.2%) | 495 (56.3%) | 558 (54.1%) |
| B cells | 488 (37.9%) | 359 (40.8%) | 377 (36.5%) |
| Immune checkpoint inhibitors / ICB | 431 (33.5%) | 340 (38.6%) | 394 (38.2%) |
| PD-1 / PD-L1 axis | 382 (29.7%) | 316 (35.9%) | 362 (35.1%) |
| Biomarkers | 334 (26.0%) | 246 (28.0%) | 294 (28.5%) |
| Spatial profiling | 223 (17.3%) | 170 (19.3%) | 194 (18.8%) |
| Single-cell analysis | 181 (14.1%) | 121 (13.8%) | 150 (14.5%) |

Values are records, n (%), within each database-specific 2000–2025 subset. One prespecified human-readable term dictionary was applied to normalized title-plus-abstract text. Each record was counted at most once per topic; topics were non-mutually exclusive. Percentages are descriptive and do not imply record-level equivalence or validated classification accuracy. These title/abstract term frequencies are distinct from the Author Keywords/Keywords Plus source-string totals reported in Table 6 and from the VOSviewer node-occurrence values shown in Figure 5A.

Supplementary Methods S1. Full PubMed and Scopus search strategies and procedures used for cross-database comparison.

PubMed and Scopus were searched independently using database-specific syntax adapted from the same conceptual framework as WoSCC. Annual counts used the WoSCC PY field, PubMed DP year, and Scopus Year, with one eligible record counted once in its database-specific publication year. Both the annual and term-profile comparisons were restricted to complete calendar years 2000–2025. For the thematic comparison, titles and abstracts were normalized by Unicode NFKC conversion, lowercasing, conversion of Unicode dash variants to the ASCII hyphen, and whitespace consolidation. The prespecified term dictionary in Supplementary Methods S2 was applied using case-insensitive whole-word or phrase matching, with the listed singular/plural and hyphen/space variants treated as equivalent; title alone was used when an abstract was unavailable. A record could contribute to several non-mutually-exclusive categories but no more than once within a category. The denominator was the corresponding database-specific complete-year subset, and frequency was calculated as matched records divided by all eligible records in that subset × 100. The datasets were analyzed separately without pooling, matching, overlap analysis, or cross-database deduplication. The rule-based term profiles are descriptive and were not treated as an accuracy-validated classifier or as formal external validation.

**Scopus search strategy**

TITLE-ABS-KEY ( ( "tertiary lymphoid structure" OR "tertiary lymphoid structures" OR "tertiary lymphoid organ" OR "tertiary lymphoid organs" OR "ectopic lymphoid structure" OR "ectopic lymphoid structures" OR "ectopic lymphoid organ" OR "ectopic lymphoid organs" OR "tertiary lymphoid aggregate" OR "tertiary lymphoid aggregates" OR "tertiary lymphoid neogenesis" OR "ectopic lymphoid neogenesis" ) AND ( immunotherap* OR "cancer immunotherapy" OR "cancer immunotherapies" OR "tumor immunotherapy" OR "tumor immunotherapies" OR "tumour immunotherapy" OR "tumour immunotherapies" OR "immune checkpoint" OR "immune checkpoints" OR "immune checkpoint blockade" OR "checkpoint blockade" OR "immune checkpoint inhibitor" OR "immune checkpoint inhibitors" OR "PD-1" OR PD1 OR "PD-L1" OR PDL1 OR "CTLA-4" OR CTLA4 OR "adoptive cell therapy" OR "adoptive cell transfer" OR "CAR-T" OR "CAR T" OR "chimeric antigen receptor" OR vaccin* OR "cancer vaccine" OR "cancer vaccines" OR "oncolytic virus" OR "oncolytic viruses" OR "oncolytic virotherapy" ) AND ( cancer* OR tumor* OR tumour* OR neoplasm* OR malignan* OR carcinoma* ) ) AND PUBYEAR > 1999 AND PUBYEAR < 2027 AND ( LIMIT-TO ( DOCTYPE , "ar" ) OR LIMIT-TO ( DOCTYPE , "re" ) ) AND ( LIMIT-TO ( LANGUAGE , "English" ) ) AND ( LIMIT-TO ( SRCTYPE , "j" ) )

**PubMed search strategy**

("tertiary lymphoid structure"[Title/Abstract] OR "tertiary lymphoid structures"[Title/Abstract] OR "tertiary lymphoid organ"[Title/Abstract] OR "tertiary lymphoid organs"[Title/Abstract] OR "ectopic lymphoid structure"[Title/Abstract] OR "ectopic lymphoid structures"[Title/Abstract] OR "ectopic lymphoid organ"[Title/Abstract] OR "ectopic lymphoid organs"[Title/Abstract] OR "tertiary lymphoid neogenesis"[Title/Abstract] OR "ectopic lymphoid neogenesis"[Title/Abstract]) AND ("Immunotherapy"[Mesh] OR "Immune Checkpoint Inhibitors"[Mesh] OR "Immunotherapy, Adoptive"[Mesh] OR immunotherap*[Title/Abstract] OR "cancer immunotherapy"[Title/Abstract] OR "cancer immunotherapies"[Title/Abstract] OR "tumor immunotherapy"[Title/Abstract] OR "tumor immunotherapies"[Title/Abstract] OR "tumour immunotherapy"[Title/Abstract] OR "tumour immunotherapies"[Title/Abstract] OR "immune checkpoint"[Title/Abstract] OR "immune checkpoints"[Title/Abstract] OR "immune checkpoint blockade"[Title/Abstract] OR "checkpoint blockade"[Title/Abstract] OR "immune checkpoint inhibitor"[Title/Abstract] OR "immune checkpoint inhibitors"[Title/Abstract] OR "PD-1"[Title/Abstract] OR PD1[Title/Abstract] OR "PD-L1"[Title/Abstract] OR PDL1[Title/Abstract] OR "CTLA-4"[Title/Abstract] OR CTLA4[Title/Abstract] OR "adoptive cell therapy"[Title/Abstract] OR "adoptive cell transfer"[Title/Abstract] OR "CAR-T"[Title/Abstract] OR "CAR T"[Title/Abstract] OR "chimeric antigen receptor"[Title/Abstract] OR vaccine*[Title/Abstract] OR "cancer vaccine"[Title/Abstract] OR "cancer vaccines"[Title/Abstract] OR "oncolytic virus"[Title/Abstract] OR "oncolytic viruses"[Title/Abstract] OR "oncolytic virotherapy"[Title/Abstract]) AND ("Neoplasms"[Mesh] OR cancer*[Title/Abstract] OR tumor*[Title/Abstract] OR tumour*[Title/Abstract] OR neoplasm*[Title/Abstract] OR malignan*[Title/Abstract] OR carcinoma*[Title/Abstract]) AND ("2000/01/01"[Date - Publication] : "2026/03/31"[Date - Publication]) AND english[Language] AND ("journal article"[Publication Type] OR "review"[Publication Type]) NOT "Published Erratum"[Publication Type] NOT Erratum[Title] NOT "Retracted Publication"[Publication Type] NOT editorial[Publication Type] NOT comment[Publication Type] NOT letter[Publication Type]

Supplementary Methods S2. Prespecified title/abstract term dictionary and accepted lexical variants.

The table below presents the human-readable term groups used for the 12 descriptive topic categories. Matching was applied to the normalized title-plus-abstract text described in Supplementary Methods S1. Terms were matched case-insensitively as whole words or phrases; the listed singular/plural and hyphen/space variants were treated as equivalent. A record could match more than one topic but was counted no more than once within any single topic.

| **Prespecified topic** | **Included terms and accepted variants** |
| --- | --- |
| **Tertiary lymphoid structures** | tertiary or ectopic lymphoid structure(s), organ(s), aggregate(s), tissue(s), or neogenesis; lymphoid neogenesis. |
| **Immunotherapy** | immunotherapy, immunotherapies, immunotherapeutic(s); immune therapy or immune-based therapy; adoptive therapy, adoptive transfer, adoptive cell therapy, or adoptive cell transfer; CAR-T/CAR T and CAR-T cell(s); chimeric antigen receptor (with or without T); cancer vaccine(s) or cancer vaccination; oncolytic virus(es) or oncolytic virotherapy; immune checkpoint inhibitor(s) or immune checkpoint blockade. |
| **Therapeutic response** | therapeutic, treatment, clinical, pathologic/pathological, radiologic/radiological, objective, or durable response(s); response to immunotherapy, treatment, therapy, immune-checkpoint therapy, PD-1, PD-L1, or CTLA-4; responder(s) or non-responder(s); sensitivity, resistance, or resistant to those treatments; treatment-sensitive, treatment-resistant, or treatment-refractory forms; complete, partial, objective, or pathologic/pathological tumor response. |
| **Prognosis / survival** | prognosis or prognostic; survival; progression-free, disease-free, recurrence-free, or relapse-free survival; clinical outcome(s). |
| **Tumor microenvironment** | tumor/tumour microenvironment; tumor/tumour immune microenvironment. |
| **T cells** | T cell(s) or T-cell(s); T lymphocyte(s); CD4 or CD8 T cell(s), including forms written with "+" or "positive". |
| **B cells** | B cell(s) or B-cell(s); B lymphocyte(s); CD19 or CD20 B cell(s), including forms written with "+" or "positive". |
| **Immune checkpoint inhibitors / ICB** | immune checkpoint inhibitor(s), inhibition, or blockade; checkpoint blockade; ICI, ICIs, or ICB; PD-1, PD-L1, or CTLA-4 inhibitor(s), inhibition, blockade, or therapy; anti-PD-1, anti-PD-L1, or anti-CTLA-4. |
| **PD-1 / PD-L1 axis** | PD-1/PD1; PD-L1/PDL1; PDCD1; CD274; programmed death 1, programmed cell death 1, programmed death-ligand 1, or programmed cell death ligand 1. |
| **Biomarkers** | biomarker(s); predictive, prognostic, or diagnostic marker(s). |
| **Spatial profiling** | spatial or spatially resolved profiling, analysis, mapping, characterization/characterisation, organization, architecture, or distribution; spatial transcriptomics, proteomics, multi-omics, or omics; multiplex(ed) immunofluorescence, immunohistochemistry, or imaging; imaging mass cytometry; digital spatial profiling; GeoMx. |
| **Single-cell analysis** | single-cell or single-cellular; single-nucleus; scRNA-seq (including hyphen/spacing variants); snRNA-seq (including hyphen/spacing variants). |
